# Education and Alcohol Consumption among Older Americans; Black–White Differences

**DOI:** 10.3389/fpubh.2016.00067

**Published:** 2016-04-21

**Authors:** Shervin Assari, Maryam Moghani Lankarani

**Affiliations:** ^1^Department of Psychiatry, University of Michigan, Ann Arbor, MI, USA; ^2^Center for Research on Ethnicity, Culture, and Health, School of Public Health, University of Michigan, Ann Arbor, MI, USA; ^3^Medicine and Health Promotion Institute, Tehran, Iran

**Keywords:** population groups, ethnic groups, African-Americans, socioeconomics, education, alcohol, drinking

## Abstract

**Purpose:**

Although the link between education and alcohol consumption is known, limited information exists on racial differences in this link. We conducted the current study to test Black–White differences in the association between education and alcohol consumption among older adults in the U.S.

**Methods:**

This cross-sectional survey enrolled 1,493 Black (*n* = 734) and White (*n* = 759) older adults (age 66 or more) in U.S. Data came from the Religion, Aging, and Health Survey, 2001. Race, demographics, socioeconomics, and alcohol consumption were measured. Independent variable was education level. Outcome was alcohol consumption. Race was the focal moderator. Logistic regression was used for data analysis.

**Results:**

Education was positively associated with ever drinking in the pooled sample. However, race interacted with education level on drinking, suggesting a smaller effect of education on drinking for Blacks compared to Whites. Among Whites, high-school graduation and college graduation were associated with increased odds of ever drinking, net of covariates. Among Blacks, high-school graduation, but not college graduation, was associated with ever drinking.

**Conclusion:**

Blacks and Whites differ in how socioeconomic status (i.e., education) shapes behaviors, especially health behaviors (i.e., drinking). How race modifies consequences and correlates of social determinants of health is not yet clear. College graduation may result in the same level of change to the social network and income of race group members. Weaker effect of education on health of Blacks may be due to the structural role of race and racism that has resulted in lower job availability and pay for Blacks.

## Introduction

Alcohol research literature has shown a U-shaped relationship between alcohol consumption and mortality risk, suggesting that alcohol consumption is associated with desired and undesired health outcomes, depending on the pattern of use. While compared with those who rarely or never drink, consuming up to one drink per day reduces the mortality risk (1); heavy alcohol use is a major risk factor for premature mortality (2, 3). Among older adults, low-to-moderate alcohol consumption also reduces overall mortality (4). By documenting the most favorable mortality profile for the individuals who consume two to six drinks per week and the most unfavorable mortality profile for those who consume two or more drinks per day, Camargo et al. concluded that “the difference between consumption of small and large amounts of alcohol consumption means the difference between preventing and causing excess mortality” (5).

Alcohol-related mortality is more common among lower socioeconomic status (SES) groups (2). Alcohol-related deaths are in fact major contributors to the SES disparities in premature mortality (3, 6). As differences between SES groups in alcohol-related mortality are larger than the differences in the pattern of use, SES is believed to be a vulnerability factor for the effects of alcohol use (7). That is low SES populations have higher vulnerability to the undesired outcomes associated with excessive alcohol use (7).

Drinking patterns and drinking outcomes are both shaped by the individuals’ SES (8–10). Education (11), employment (12), and financial capacity (13) determine pattern and frequency of alcohol use. Most studies have documented positive effects of education and income on risk of being an ever or current drinker. Compared to abstainers and heavy drinkers, moderate drinkers tend to enjoy higher SES and less frequently suffer alcohol-related problems (8–13).

Similar to many other parts of the world, SES is a major social determinant of drinking in American society (14, 15). In the U.S., the proportion of current drinkers increases with education and income (15), with some research reporting a U-shaped relationship between income and average daily alcohol consumption (16). Compared with consistent drinkers, consistent abstainers have lower educational levels, and lower incomes are more often unemployed. Greater alcohol consumption is also associated with having a higher educational level and having a higher income (17). Lower income predicts a larger decline in drinking levels over time among Americans (17). In the college sample, educational achievement is positively associated with drinking (18). While inconsistent findings have been also reported (16), most studies suggest that lower SES groups drink heavier quantities, while higher SES groups drink more frequently but less quantity (10).

Race and ethnicity also influences the pattern of alcohol use in the U.S. (14). While Blacks begin regular alcohol use later than Whites, they move more rapidly from initiation of regular alcohol use to problem use, a phenomenon called telescoping (14). In the U.S., the proportion of current drinkers is lower than average among Black adults (15). While consistent abstainers are more frequently Blacks, more Whites are consistent drinkers than Blacks (19). According to a study in the U.S. compared to Blacks, Whites have a higher tendency to drink at restaurants, bars, clubs, or organizational meetings. Blacks more frequently than Whites drink in public settings such as parks, streets, and parking lots (20). Latent class analysis of 4,646 older current drinkers from the National Epidemiologic Survey on Alcohol and Related Conditions showed that Blacks had lower prevalence of being a moderate-risk drinker (21). At the same time, Blacks are more likely to be in the high-risk drinking class, if they drink (21), and older Black alcoholics suffer greater undesired medical and psychosocial outcomes (22).

In the U.S., race is one of the main determinants of employment opportunities and employer choices (23–25). Structural inequalities due to race exist in the labor market preferences in the U.S. (26). Sociology and economics literature has shown wage inequality (27) and occupational segregation based on race. Particularly for individuals with the highest levels of education, differences in average income of Blacks and Whites reflect a huge racial gap in how education is being translated to health of minorities in the U.S. (28).

In addition to different employment opportunity, profits for equal jobs are different for Blacks and Whites, a phenomenon called Black–White pay gaps (29). Some of the limited job opportunities for Blacks are also due to racial residential segregation, which is against job proximity for Black neighborhoods (30). Lower school quality where Blacks live is another cause for differential effect of schooling on wages and employment opportunities (31). For example, in 2006, Black men with a master’s degree earned $27,000 less than Whites with the same education (28).

The protective effect of education on health is conditional on the availability of other resources – such as income and wealth – which are under influence of race (32–34).^1^ The ability of an individual to translate their socioeconomic resources into better health depends on demographic characteristics such as race (35–38). In other words, race and social class alter the health gain associated with education attainment (35–39).

Race changes the health effects of education (40). Williams argued that the effects of race and SES on mortality are not additive, but multiplicative (35). Thus, health should be conceptualized as the outcome of the interaction between race and a wide range of factors, such as education and income, that almost always place Blacks at more social and economic disadvantages (35). Our findings showed that race changes the effect of education on drinking, which in line with previous research suggests that the effects of race and SES are multiplicative other than additive.^1^

Based on the differential effect hypothesis (41), correlates of psychosocial and behaviors factors – that ultimately shape health and illness of population – are not universal but specific to populations. According to this theory, effects of risk and protective factors are shaped by a wide range of contextual factors such as race, ethnicity, gender, and place. Similar to other behaviors, drinking also has group-specific antecedents and consequences, which vary among Whites and Blacks (42–51). Using this conceptual model, we conducted the current study to compare Blacks and Whites for the association between education attainment and drinking behavior of older adults. To provide results that are generalizable to the U.S., we used a nationally representative cohort of older Americans.

## Materials and Methods

### Design and Setting

This was a cross-sectional study with 3 years of follow-up data. Data came from Wave 1 of the Religion, Aging, and Health Survey, 2001, a nationally representative household survey of Black and White older adults in the U.S. (52).

### Ethics

The study protocol was approved by the University of Michigan Institutional Review Board (IRB). All participants provided consent. Data were collected anonymously.

### Sampling and Participants

The study only included White or Black older adults. All participants were non-institutionalized English-speaking people of age more than 65 years. The study population was limited to Christians or those who were never associated with any faith. Older Blacks were oversampled in the survey.

### Measures

Age (continuous measure), gender (female = 1, male = 0), race (Blacks = 1, Whites = 0), income (continuous measure), education (a three-level ordinal variable), and drinking (binary outcome) were measured.

#### Education

Following items were used to measure education: (1) did you get a high-school diploma or pass the high-school equivalency test? and (2) did you graduate from college and receive a bachelor’s degree? We treated our education variable as a three-level categorical variable: (0) less than high-school diploma [reference group], (1) completed high-school diploma, and (2) graduated from college.

#### Income

Participants were asked: here is a card showing amounts of yearly income. Into which of these groups did (your/your family’s) total income, from all sources, fall last year, 2000, before taxes? Responses included (1) less than $5,000, (2) $5,001–$9,999, (3) $10,000–$14,999, (4) $15,000–$19,999, (5) $20,000–$24,999, (6) $25,000–$29,999, (7) $30,000–$39,999, (8) $40,000–$59,999, (9) 60,000–$79,999, and (10) $80,000+. This variable was operationalized as a continuous measure, ranging from 1 to 10.

#### Drinking

Three items were used to assess drinking behavior. Items included (1) do you ever drink beer, wine, or liquor?, (2) during the last month, on how many days did you drink beer, wine, or liquor?, and (3) on the days you drink, how many cans of beer, glasses of wine, or drinks of liquor do you usually have? Our main outcomes were ever drinking, current drinking (last month), and problem drinking (5+ drinks per day), treated as three separate binary outcomes. Similar items have been shown to provide valid and reliable measures to study drinking patterns (53–55).

### Statistical Analysis

We used SPSS 20.0 for data analysis. We fitted logistic regressions in the pooled sample (IBM Inc., Armonk, NY, USA), and specific to race, with education (socioeconomics) as the independent variable, ever drinking as the outcome, demographics (age and gender) as the covariates, and race as the focal moderator. We did not fit models for problem drinking as outcome, due to very limited number of individuals who reported five or more drinks per day. Odds ratios with 95% confidence intervals (CI) were reported.

## Results

The study enrolled 1,493 older individuals (age 65 or more). This number is composed of Blacks (*n* = 734) and Whites (*n* = 759). Table 1 presents descriptive statistics overall and also based on race. While age was not significantly different between Blacks and Whites, Blacks were more likely female, had lower education, were less frequently married, more frequently smoked, and less frequently reported ever drinking.

**Table 1:** **Descriptive statistics for the analytic sample, stratified by race and overall**.

	All		Whites		Blacks	
	Mean	SD	Mean	SD	Mean	SD
Age	75.14	6.66	75.37	6.82	74.91	6.49
	***n***	**%**	***n***	**%**	***n***	**%**
Gender*						
Male	570	38.2	314	41.4	256	34.9
Female	923	61.8	445	58.6	478	65.1
Education (high-school diploma)						
Yes	872	59.0	552	73.4	320	44.0
No	607	41.0	200	26.6	407	56.0
Education (college)						
Yes	872	59.0	552	73.4	320	44.0
No	607	41.0	200	26.6	407	56.0
Marital status (married)*						
No	773	52.2	306	40.5	467	64.3
Yes	708	47.8	449	59.5	259	35.7
Current smoking*						
Yes	155	10.4	60	7.9	95	13.0
No	1,336	89.6	698	92.1	638	87.0
Ever drinking*						
No	1,316	88.4	694	91.8	622	84.9
Yes	173	11.6	62	8.2	111	15.1
Current drinking*						
No	1,182	78.8	553	72.9	624	85.0
Yes	318	21.2	206	27.1	110	15.0
Problem drinking						
No	1,486	99.1	752	99.1	727	99.0
Yes	14	0.9	7	0.9	7	1.0

*P < 0.05.

Table 2 shows the results of logistic regressions in the pooled sample, with and without the interaction terms. Higher education was associated with higher odds of ever drinking. We also found a significant interaction between race and education level on odds of ever drinking, suggesting a smaller effect of higher education on drinking for Blacks compared to Whites.

**Table 2:** **The association between baseline high-school graduation and college graduation with drinking in the pooled sample (*n* = 1,439)**.

	Model 1			Model 2		
	OR (SE)	95% CI	*P*	OR (SE)	95% CI	*P*
Age	0.98 (0.01)	0.96–0.99	0.044	0.98 (0.01)	0.95–0.99	0.038
Gender (female)	0.31 (0.15)	0.23–0.42	0.000	0.32 (0.15)	0.23–0.43	0.000
Race (Blacks)	0.63 (0.16)	0.46–0.86	0.004	0.73 (0.26)	0.43–1.22	0.225
Education (high-school diploma)						
No diploma	Ref			Ref		
High-school diploma	1.70 (0.18)	1.20–2.40	0.003	1.76 (0.25)	1.08–2.87	0.024
College	2.38 (0.26)	1.44–3.92	0.001	3.10 (0.31)	1.68–5.71	0.000
Marital status (married)	0.80 (0.17)	0.57–1.12	0.187	0.81 (0.17)	0.58–1.13	0.210
Income	1.17 (0.04)	1.09–1.27	0.000	1.18 (0.04)	1.09–1.27	0.000
High-school diploma × Blacks				0.98 (0.34)	0.51–1.89	0.951
College × Blacks				0.35 (0.53)	0.12–0.98	0.046

Table 3 provides a summary of the associations between education and ever drinking among Blacks and Whites. Among Whites, high-school graduation and college graduation were both associated with increased odds of ever drinking, net of covariates. Compared to lowest education level, high-school graduation was associated with higher odds of ever drinking. The same association could not be found for college graduation among Blacks (Table 3).

**Table 3:** **The association between baseline high-school graduation and college graduation with drinking among Whites (*n* = 759) and Blacks (*n* = 734)**.

	Whites			Blacks		
	OR (SE)	95% CI	*P*	OR (SE)	95% CI	*P*
Age	0.97 (0.01)	0.95–1.002	0.071	0.98 (0.02)	0.94–1.014	0.225
Gender (female)	0.42 (0.20)	0.29–0.628	<0.001	0.20 (0.25)	0.13–0.334	<0.001
Education (high-school diploma)			0.007			0.024
No diploma	Ref			Ref		
High-school diploma	1.61 (0.25)	0.98–2.629	0.059	1.98 (0.25)	1.21–3.246	0.007
College	2.75 (0.32)	1.47–5.142	0.002	1.41 (0.51)	0.52–3.798	0.495
Marital status (married)	0.79 (0.23)	0.51–1.224	0.286	0.79 (0.26)	0.47–1.323	0.370
Income	1.22 (0.05)	1.11–1.347	<0.001	1.10 (0.07)	0.97–1.257	0.145

## Discussion

Higher education was associated with higher odds of ever drinking among American older adults. Education level interacted with race on odds of ever drinking, suggesting a smaller effect of highest education level on ever drinking for Blacks compared to Whites. While among Whites, high-school graduation and college graduation were both associated with increased odds of ever drinking, high-school graduation, but not college graduation, was associated with higher odds of ever drinking among Blacks.

Although Mirowsky and Ross have described the effect of education on health as “enduring, consistent, and growing” (56), there are Black–White differences in the effect of education on health. In a study by Hayward et al., Whites showed an effect of education on mortality, while there was almost no association for Blacks (57). Backlund et al. found that while for Whites, each additional year of education had a stronger effect on decreasing mortality for those with a high-school diploma, for Blacks, there were step reductions in mortality at 12 and 16 years of education, with constant slopes between the steps (58). Everett et al. also showed race differences in the effect of education on mortality (37).

Findings of the current study on the stronger association between highest level of education and ever drinking for White compared to Black older adults may be due to the labor market preference for Whites, which lowers the effects of education in changing life style, behaviors, social network, and social norms of Blacks (59), which is in part due to unequal opportunities in education and employment (60). Labor market discrimination against Blacks, particularly Black men, is well known (61). According to the dual market theory, jobs are roughly divided into two groups: primary jobs are those with relatively high wages, good working conditions and opportunities for advancement into higher paying jobs; secondary jobs are those with low wages, bad working conditions, unstable employment, and minimal opportunity for advancement (62). Most Blacks and other minorities work on secondary jobs, if they obtain any employment (63).

Diminishing returns hypothesis suggests that racial disparity is largest at the highest SES levels, because with each level of increase in SES level, Blacks gain less improvement in health than Whites (64). Racial differences are not the same at low versus high SES levels (65). College attendance has shown to have a smaller effect on changing the life circumstances and behaviors of Blacks, as they less frequently afford to leave town to go to college (66). Similar to the previous research (32–34, 67) showing group differences in the health benefits of education, Assari et al. and others (40) found that the protective effects of education against mortality may not be uniform across population groups.

This study helps us understand how race and ethnicity alter how SES, behaviors, and health cluster (41–51). A growing literature has documented weaker role of a number of psychosocial factors, such as depressive symptoms, SRH, hostility, and other psychosocial outcomes on risk of mortality of Blacks compared to Whites (68–70). Previous research has shown that race and ethnicity alter the effects of SES on mortality (71–73). Race-specific role of SES may be a consequence of racial differences in distribution of risk and protective factors that influence physical health and mental health. Thus, SES may differently represent vulnerabilities to the effects of risk and protective factors (68–71). In addition, contextual factors, such as race and ethnicity, shape additive effects of SES, depression, and physical health even when their separate effects are similar (49, 74). Finally, psychosocial and subjective health indicators do not necessarily reflect same aspects of health across populations (74, 75).

Our study is subject to a number of limitations. First, we used a cross-sectional design, which does not allow causal inferences. There is a need for additional longitudinal studies that follow individuals over time. Second, drinking was measured using three single-item measures, not a standardized scale. We do not have information on alcohol use-related disorders (e.g., abuse or dependence) that require diagnosis by a clinician or detailed structured interview. Third, although some covariates were controlled, we did not control for other potential covariates, including health, function, mood, and access to medical care. Lack of data on cognitive function was another limitation. This is particularly important given the protective effects of light-to-moderate alcohol consumption against dementia and cognitive decline in older adults (76–78). Despite the above limitations, this is one of the first studies to document Black–White differences in the role of SES on drinking behavior of older adults in the U.S. Using a representative sample was the other strength of this study. Our findings have implications for racial health disparities in the U.S., given the higher burden of alcohol-related problems among Blacks (79).

Overall, our findings suggest that Black–White differences exist in the association between college graduation and drinking among older adults in the U.S. Further research is needed to better understand the mechanisms behind race and ethnic differences in behavioral correlates of SES.

## Ethics

All procedures followed were in accordance with the ethical standards of the responsible committee on human experimentation (institutional and national) with the Helsinki Declaration of 1975, as revised in 2000. Informed consent was obtained from all participants who were included in the study. University of Michigan Institutional Review Board (IRB) approved the study protocol.

## Informed Consent

All procedures followed were in accordance with the ethical standards of the responsible committee on human experimentation (institutional and national) with the Helsinki Declaration of 1975, as revised in 2000. Informed consent was obtained from all participants who were included in the study.

## Author Contributions

SA designed and analyzed this work, and contributed to draft and revision. ML contributed to design and revised the paper. All authors confirmed the last version.

## Conflict of Interest Statement

The authors declare that the research was conducted in the absence of any commercial or financial relationships that could be construed as a potential conflict of interest.

## Footnotes

^1^Assari S, Racial disparities in the link between education and mortality in United States. JAMA. (Under review, 2016).
